# Can Human Embryonic Stem Cell-Derived Stromal Cells Serve a Starting Material for Myoblasts?

**DOI:** 10.1155/2017/7541734

**Published:** 2017-06-15

**Authors:** Yu Ando, Marie Saito, Masakazu Machida, Chikako Yoshida-Noro, Hidenori Akutsu, Masataka Takahashi, Masashi Toyoda, Akihiro Umezawa

**Affiliations:** ^1^Center for Regenerative Medicine, National Research Institute for Child Health and Development, Tokyo 157-8535, Japan; ^2^Department of Applied Molecular Chemistry, College of Industrial Technology, Nihon University, Tokyo, Japan; ^3^Research Team for Geriatric Medicine (Vascular Medicine), Tokyo Metropolitan Institute of Gerontology, Tokyo 173-0015, Japan

## Abstract

A large number of myocytes are necessary to treat intractable muscular disorders such as Duchenne muscular dystrophy with cell-based therapies. However, starting materials for cellular therapy products such as myoblasts, marrow stromal cells, menstrual blood-derived cells, and placenta-derived cells have a limited lifespan and cease to proliferate in vitro. From the viewpoints of manufacturing and quality control, cells with a long lifespan are more suitable as a starting material. In this study, we generated stromal cells for future myoblast therapy from a working cell bank of human embryonic stem cells (ESCs). The ESC-derived CD105^+^ cells with extensive in vitro proliferation capability exhibited myogenesis and genetic stability in vitro. These results imply that ESC-derived CD105^+^ cells are another cell source for myoblasts in cell-based therapy for patients with genetic muscular disorders. Since ESCs are immortal, mesenchymal stromal cells generated from ESCs can be manufactured at a large scale in one lot for pharmaceutical purposes.

## 1. Introduction

Duchenne muscular dystrophy is an intractable genetic disorder, and effective therapies have not yet been developed. Novel approaches to treat Duchenne muscular dystrophy include small molecules, gene therapy, and biologics such as cytokines and cell-based therapy [1, 2]. Among these advanced therapeutic approaches, regenerative therapies have been focused due to the recent advances of pluripotent stem cells with different types of reprogramming technologies [3, 4]. In vitro expansion of quality-controlled stem cells and transplantation into patients with degenerative diseases in an allogeneic manner can be one of the ideal therapeutic scenarios. Somatic cells such as myoblasts, marrow stromal cells, menstrual blood-derived cells, and placenta (amnion, cholate plate, umbilical cord)-derived cells have been introduced as starting materials for cellular therapy products [5–9]. However, these somatic cells have a limited lifespan and cease to proliferate in vitro, and thus, sufficient numbers of cells cannot be prepared to treat muscles of a whole body in cell-based therapies. From this viewpoint, cells with a long lifespan are more suitable for starting materials.

Human pluripotent stem cells such as embryonic stem cells (ESCs) and induced pluripotent stem cells (iPSCs) are immortal and can therefore be a good source of large number of cellular therapy products with one lot for genetic muscular disorders [1]. In addition to immortality, ESCs and iPSCs exhibit pluripotency, that is, capability to differentiate theoretically into almost all types of cells including myoblasts and their progenitor cells [10]. As therapeutic cellular products, myoblasts and mesenchymal stromal cells are considered the most suitable. In this study, we generated mesenchymal stromal cells from ESCs for the production of cellular therapy products to treat patients with genetic muscular disorders [11, 12]. We developed a novel protocol to manufacture mesenchymal stromal cells from ESCs with certified materials that had been analyzed for viruses.

## 2. Results

### 2.1. Generation of Mesenchymal Stromal Cells

**Figure 1:**
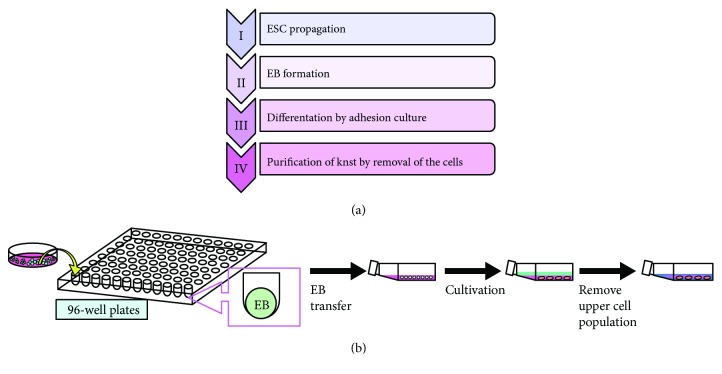
Generation of mesenchymal stromal cells from sees2. (a) Step-by-step manufacturing process. (b) Scheme for generation of mesenchymal stromal cells from sees2.

To generate mesenchymal stromal cells from human ESCs, we propagated sees2 cells on mouse embryonic fibroblasts (MEFs) and formed embryoid bodies (EBs) for 4 days on a feeder layer of freshly plated gamma-irradiated mouse embryonic fibroblasts (Figure 1). The EBs were then transferred to the collagen-coated flasks and cultivated for 60 to 70 days. The upper adherent cell layer was detached to obtain a resource of mesenchymal stromal cells.

### 2.2. Propagation of Mesenchymal Stromal Cells

**Figure 2:**
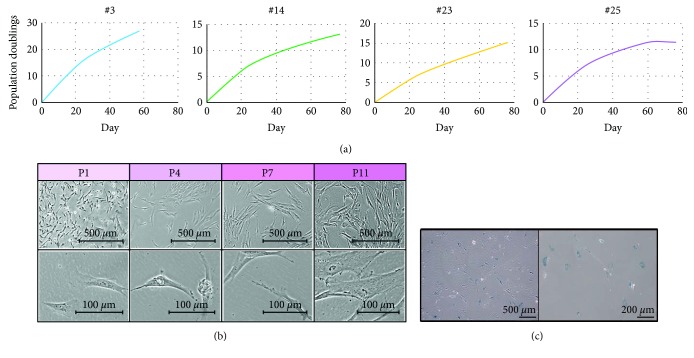
Characterization of mesenchymal stromal cells. (a) Growth curve of mesenchymal stromal cells (knst#3, #14, #23, #25). (b) Phase contrast photomicrography of mesenchymal stromal cells (knst#2: passages 1, 4, 7, and 11). (c) Senescence-associated beta-galactosidase stain (knst#3, passage 11).

We repeated generation of mesenchymal stromal cells from sees2 cells in 4 different independent experiments (#3, #14, #23, and #25) and investigated proliferation rate of the mesenchymal stromal cells (#3, #14, #23, and #25) for over 50 days (Figure 2(a)). The mesenchymal stromal cells rapidly proliferated in culture and propagated continuously, however stopped replicating, became broad and flat, and exhibited SA-*β*-galactosidase activity as indicated by blue staining of their cytoplasm at passage 11 (Figures 2(b) and 2(c)). The enlargement of the cell size was passage-dependent.

### 2.3. Flow Cytometric and Karyotypic Analysis

**Figure 3:**
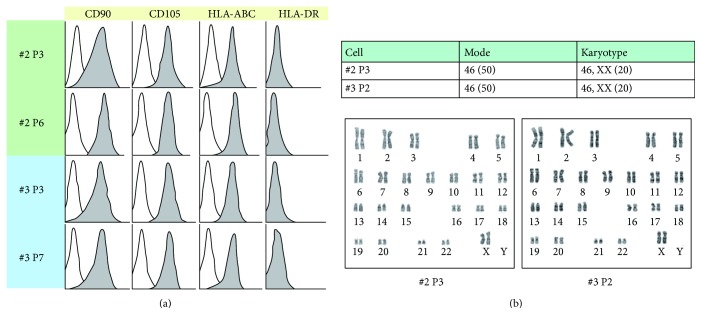
Flow cytometric analysis and karyotypic analysis. (a) Flow cytometric analysis of knst#2 (passages 3 and 6) and knst#3 (passages 3 and 7). (b) Karyotypic analysis of knst#2 (passage 3) and knst#3 (passage 2).

Flow cytometric analysis revealed that the mesenchymal stromal cells #2 and #3 were positive for CD90, CD105, and HLA-ABC and negative for HLA-DR (Figure 3(a)). The expression level and pattern of these markers remained unchanged after 3 or 4 passages (12 or 16 population doublings, resp.). Karyotypic analyses of the mesenchymal stromal cells #2 and #3 were performed at passages 3 and 2, respectively (Figure 3(b)). They were found to be diploid and not to exhibit any significant abnormalities. The chromosome number of both #2 and #3 was 46 without exception.

### 2.4. Global Outlook by Hierarchical Clustering and Principal Component Analysis (PCA)

**Figure 4:**
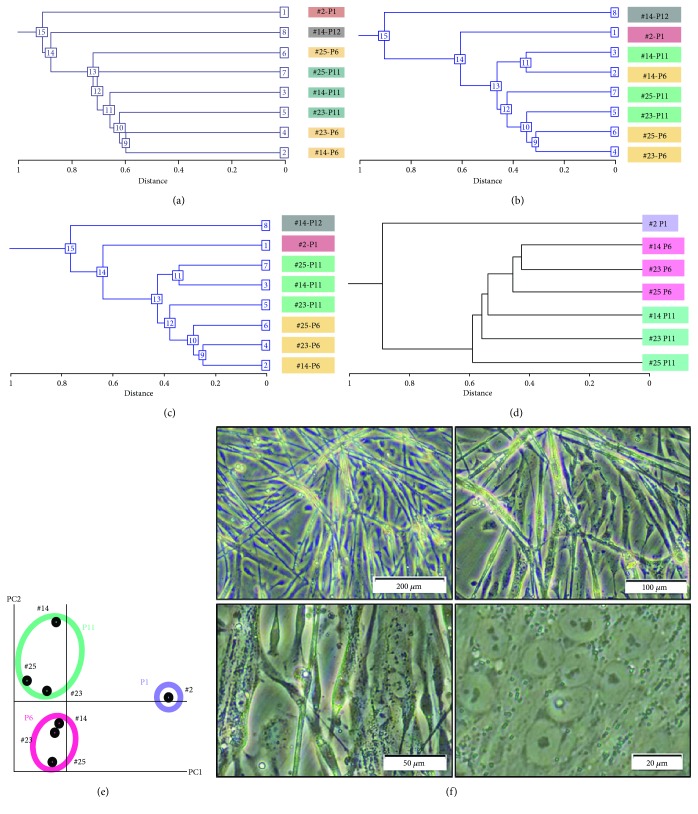
Global gene expression analysis of ESC-derived mesenchymal stromal cells. (a) Hierarchical clustering analysis based on the expression of all genes (58,201 probes on an Agilent SurePrint G3 Human GE v3 8x60K Microarray). (b) Hierarchical clustering analysis based on expression levels of the mesenchyme-associated genes. (c) Hierarchical clustering analysis based on expression levels of the stem cell-associated genes. (d) Hierarchical clustering analysis based on expression levels of the muscle-associated genes. (e) Principal component analysis of the muscle-associated genes. (f) Phase-contrast photomicrographs of knst myogenesis.

To investigate myogenic potential, mesenchymal stromal cells were analyzed, depending on gene expression levels. Hierarchical clustering analysis based on all probes, mesenchyme-associated genes, and stem cell-associated genes revealed that the mesenchymal stromal cells were categorized into the same group in a passage-dependent manner (Figures 4(a), 4(b), and 4(c)). Likewise, hierarchical clustering analysis and PCA on the expression pattern of the myogenic and cardiomyogenic genes also show passage-dependent categorization (Figures 4(d) and 4(e), Supplemental Table 1 available online at https://doi.org/10.1155/2017/7541734). After the induction, the mesenchymal stromal cells started to form multinucleated myotubes (Figure 4(f)).

## 3. Discussion

For the development of cell-based therapeutic strategies to genetic myogenic disorders, immortal cells as a raw material are required to gain sufficient number of cells, and detailed studies are therefore essential with regard to the characteristics of differentiated mesenchymal stromal cells. This present study demonstrated the detailed alterations of the mesenchymal stromal cells during expansion from P0 to P11 in monolayer culture. The fate of mesenchymal stromal cells generated from ESCs depended on passage number or population doubling levels in culture. In our previous study, we showed that human marrow stromal cells and umbilical cord blood-derived cells reach senescence, exhibit large, flat morphology at late passages, and have different characteristics, depending on passage number and population doublings [13, 14]. Myogenic ability of ESC-derived mesenchymal stromal cells is possibly associated with surface markers, morphology, cytokines, and differentiation capacity. c-kit, CD34, and CD140 serve as good markers to distinguish murine mesenchymal cells with multipotency, that is, mesenchymal stem cells [15]. CD29+, CD44+, CD59+, and CD90+ cells from menstrual blood are capable of differentiating into myoblasts/myocytes and conferring human dystrophin expression in the murine model for Duchenne muscular dystrophy [6]. In this study, we generated high-purity mesenchymal stromal cells for future myoblast therapy from a working cell bank of ESCs. The ESC-derived CD105+ cells with in vitro extensive proliferation capability exhibited myogenesis and genetic stability in vitro, implying that ESC-derived CD105+ cells are another cell sources for myoblasts in cell-based therapy to patients with genetic muscular disorders. Since ESCs are immortal, mesenchymal stromal cells generated from ESCs can be manufactured in a large scale with one lot in pharmaceutical purpose.

Mesenchymal stromal cells derived from ESCs have been examined from the viewpoints of differentiation propensity, surface markers, proliferation, and morphology [10, 16–20]. They exhibit multipotency, that is, adipogenic, osteogenic, and chondrogenic differentiation in vitro [10]. They also show myogenic differentiation in vitro like mesenchymal stem cells derived from the bone marrow, menstrual blood, and placenta [6, 8, 13, 21–23]. These mesenchymal stromal cells can be used for therapeutic agents or delivery vehicles to patients with graft-versus-host disease, ischemic heart disease, and lysosomal storage disorders. With the robust scalable manufacturing process described in this study, ESC-derived CD105+ cells serve as a starting material of these possible cellular therapy agents. ESC-derived CD105+ cells were mortal while the original ESCs (sees2) were immortal. The cells are, therefore, nontumorigenic because they reach senescence or stop dividing after a limited number of replications. This limited cell lifespan could be an advantage from the viewpoint of tumorigenicity, but a disadvantage for scalable manufacturing of cell therapy products. iPSC-derived mesenchymal stromal cells exhibit almost the same phenotypes in differentiation propensity, surface markers, proliferation, and morphology as ESC-derived CD105+ cells [19, 24]. Taken together, there is no great distinction in quality attributes of mesenchymal stromal cells derived from ESCs, iPSCs, and various tissues.

Implantation of myoblasts induced from ESC-derived mesenchymal stromal cells into patients with genetic muscular disorders is indeed an ideal strategy, from the viewpoint of industry-based, sustainable supply of large quantities of affordable, quality-controlled cells. It is unlikely that it is possible to prepare unaffected somatic cells in sufficient quantity, necessitating the use of stem cells from suitable, cost-effective allogeneic sources, such as ESCs and iPSCs. The cellular therapy products manufactured from ESCs and iPSCs can cover whole-body muscle because of their immortality. In addition, the proliferation capability and genetic stability of the ESC-derived mesenchymal stromal cells open up significant new possibilities in regenerative medicine. ESCs can be a promising cellular source for cell-based therapy to treat Duchenne muscular dystrophy, a lethal human disease for which no effective treatment currently exists [11, 12].

## 4. Materials and Methods

### 4.1. Ethical Statement

Human cells in this study were performed in full compliance with the Ethical Guidelines for Clinical Studies. The cultivation of hESC lines were performed in full compliance with “the Guidelines for Derivation and Distribution of Human Embryonic Stem Cells (Notification of the Ministry of Education, Culture, Sports, Science, and Technology in Japan (MEXT))” and “the Guidelines for Utilization of Human Embryonic Stem Cells (Notification of MEXT).” The experimental procedures were approved by the Institutional Review Board (IRB) at the National Center for Child Health and Development. Animal experiments were performed according to protocols approved by the Institutional Animal Care and Use Committee of the National Research Institute for Child Health and Development. All experiments with mice were subject to the 3 R consideration (refine, reduce, and replace), and all efforts were made to minimize animal suffering and to reduce the number of animals used.

### 4.2. hESC Culture

sees2 and sees5 were routinely cultured onto a feeder layer of freshly plated gamma-irradiated mouse embryonic fibroblasts (MEFs), isolated from ICR embryos at 12.5 gestations, in the hESC culture media. The hESC media consisted of Knockout™-Dulbecco's modified Eagle's medium (KO-DMEM) (Life Technologies, CA, USA; number 10829-018) supplemented with 20% 35 kGy irradiated Knockout™-Serum Replacement (KO-SR; number 10828-028), 2 mM Glutamax-I (number 35050-079), 0.1 mM nonessential amino acids (NEAA; number 11140-076), 50 U/ml penicillin-50 *μ*g/ml streptomycin (Pen-Strep) (number 15070-063), and recombinant human full-length bFGF (Kaken Pharmaceutical Co. Ltd.) at 50 ng/ml. Cells were expanded using enzymatic passaging by recombinant trypsin (Roche Diagnostics, Indianapolis, USA).

### 4.3. Manufacturing Procedure

To generate EBs, sees2 and sees5 (5 × 10^3^/well) were dissociated into single cells with 0.5 mM EDTA (Life Technologies) after exposure to the rock inhibitor (Y-27632: A11105-01, Wako, Japan) and cultivated in 96-well plates (Thermo Fisher Scientific) in the EB medium (76% Knockout DMEM, 20% 35 kGy irradiated Xeno-free Knockout Serum Replacement (XF-KSR, Life Technologies, CA, USA), 2 mM GlutaMAX-I, 0.1 mM NEAA, Pen-Strep, and 50 *μ*g/ml l-ascorbic acid 2-phosphate (Sigma-Aldrich, St. Louis, MO, USA)) for 4 days. The EBs were transferred to T25 flasks coated with NMP collagen PS (Nippon Meat Packers Inc.) and cultivated in the XF32 medium (85% Knockout DMEM, 15% 35 kGy-irradiated XF-KSR, 2 mM GlutaMAX-I, 0.1 mM NEAA, Pen-Strep, 50 *μ*g/ml l-ascorbic acid 2-phosphate, 10 ng/ml heregulin-1*β* (recombinant human NRG-beta 1/HRG-beta 1 EGF domain; Wako, Japan), 200 ng/ml recombinant human IGF-1 (LONG R3-IGF-1; Sigma-Aldrich), and 20 ng/ml human bFGF (Kaken Pharmaceutical Co. Ltd.)) for 60 to 70 days. The flasks were gently shaken to detach the cells. The detached cells were aggregated and could thus be easily removed by a pipette. The remaining adherent cells in the flasks were used for a resource of mesenchymal stromal cells. The adherent cells were then propagated in *α*-MEM medium supplemented with 10% FBS (Gibco or HyClone) and 1% Pen-Strep for further in vitro analysis.

### 4.4. Karyotypic Analysis

Karyotypic analysis was contracted out to Nihon Gene Research Laboratories Inc. (Sendai, Japan). Metaphase spreads were prepared from cells treated with 100 ng/ml of colcemid (Karyo Max, Gibco Co. BRL) for 6 h. The cells were fixed with methanol:glacial acetic acid (2 : 5) three times and placed onto glass slides (Nihon Gene Research Laboratories Inc.). Chromosome spreads were Giemsa banded and photographed. A minimum of 10 metaphase spreads were analyzed for each sample and karyotyped using a chromosome imaging analyzer system (Applied Spectral Imaging, Carlsbad, CA).

### 4.5. Gene Chip Analysis

Total RNA was extracted using TRIzol reagent (Thermo Fisher Scientific Inc.) according to the manufacturer's instructions. RNA quantity and quality were determined using a Nanodrop ND-1000 spectrophotometer (Thermo Fisher Scientific Inc.) and an Agilent Bioanalyzer (Agilent Technologies, Santa Clara, CA). Total RNA was amplified and labeled with cyanine 3 (Cy3) using an Agilent Low Input Quick Amp Labeling Kit, one-color (Agilent Technologies) following the manufacturer's instructions. Briefly, total RNA was reversed transcribed to double-strand cDNA using a poly dT-T7 promoter primer. Primer, template RNA, and quality control transcripts of known concentration and quality were first denatured at 65°C for 10 min and incubated for 2 hours at 40°C with 5X first-strand buffer, 0.1 M DTT, 10 mM dNTP mix, and AffinityScript RNase Block Mix. The AffinityScript enzyme was inactivated at 70°C for 15 min. cDNA products were then used as templates for in vitro transcription to generate fluorescent cRNA. cDNA products were mixed with a transcription master mix in the presence of T7 RNA polymerase and Cy3-labeled CTP and incubated at 40°C for 2 hours. Labeled cRNAs were purified using QIAGEN's RNeasy mini spin columns and eluted in 30 *μ*l of nuclease-free water. After amplification and labeling, cRNA quantity and cyanine incorporation were determined using a Nanodrop ND-1000 spectrophotometer and an Agilent Bioanalyzer. For each hybridization, 0.60 *μ*g of Cy3-labeled cRNA were fragmented and hybridized at 65°C for 17 hours to an Agilent SurePrint G3 Human GE v3 8x60K Microarray. After washing, microarrays were scanned using an Agilent DNA microarray scanner. Intensity values of each scanned feature were quantified using Agilent feature extraction software version 11.5.1.1, which performs background subtractions. We only used features which were flagged as no errors (detected flags) and excluded features which were not positive, not significant, not uniform, not above background, saturated, and population outliers (not detected and compromised flags). Normalization was performed using Agilent GeneSpring software version 13.0 (per chip:normalization to 75 percentile shift). There are total of 58,201 probes on an Agilent SurePrint G3 Human GE v3 8x60K Microarray without control probes. Hierarchical clustering analysis and Principal Component Analysis were performed using NIA Array Analysis (https://lgsun.grc.nia.nih.gov/ANOVA/).

## Supplementary Material

Supplemental Table 1. List of the muscle-associated genes.
