# Supplementary material for: Can Human Embryonic Stem Cell-Derived Stromal Cells Serve a Starting Material for Myoblasts?
Source: Stem Cells Int. 2017 Jun 15;2017:7541734. doi: 10.1155/2017/7541734 (PMC5494578; doi:10.1155/2017/7541734)
Supplement: Supplementary file 1 — Supplemental Table 1. List of the muscle-associated genes. [file 7541734.f1.pdf]

Supplemental Table 1. List of the muscle-associated genes

Systematic	Common	Description
A_23_P368691	ABCC9	ATP-binding cassette, sub-family C (CFTR/MRP), member 9
A_24_P178503	ABCC9	ATP-binding cassette, sub-family C (CFTR/MRP), member 9
A_23_P307310	ACAN	aggrecan
A_33_P3500167	ACAN	aggrecan
A_33_P3229288	ACE	angiotensin I converting enzyme
A_23_P252981	ACE2	angiotensin I converting enzyme 2
A_33_P3236382	ACTC1	actin, alpha, cardiac muscle 1
A_23_P79221	ACVR1	activin A receptor, type I
A_33_P3360665	ACVR1	activin A receptor, type I
A_23_P374082	ADAM19	ADAM metallopeptidase domain 19
A_23_P81369	ADAM19	ADAM metallopeptidase domain 19
A_23_P49816	ADAP2	ArfGAP with dual PH domains 2
A_22_P00012098	ADRA1A	adrenoceptor alpha 1A
A_23_P8938	ADRA1A	adrenoceptor alpha 1A
A_33_P3377194	ADRA1A	adrenoceptor alpha 1A
A_33_P3310189	ADRB1	adrenoceptor beta 1
A_33_P3384392	ADRBK1	adrenergic, beta, receptor kinase 1
A_23_P115261	AGT	angiotensinogen (serpin peptidase inhibitor, clade A, member 8)
A_23_P62309	AGTR2	angiotensin II receptor, type 2
A_33_P3376493	AGTR2	angiotensin II receptor, type 2
A_23_P368126	AHCYL1	adenosylhomocysteinase-like 1
A_24_P11307	AHCYL1	adenosylhomocysteinase-like 1
A_23_P70746	AHI1	Abelson helper integration site 1
A_24_P38143	AHI1	Abelson helper integration site 1
A_24_P142095	AKAP13	A kinase (PRKA) anchor protein 13
A_23_P14351	AKAP6	A kinase (PRKA) anchor protein 6
A_23_P123108	AKAP9	A kinase (PRKA) anchor protein 9
A_23_P309261	AKAP9	A kinase (PRKA) anchor protein 9
A_33_P3380196	AKAP9	A kinase (PRKA) anchor protein 9
A_33_P3380211	AKAP9	A kinase (PRKA) anchor protein 9
A_33_P3210099	ALPK3	alpha-kinase 3
A_23_P133068	ANK2	ankyrin 2, neuronal
A_33_P3287967	ANK2	ankyrin 2, neuronal
A_23_P161218	ANKRD1	ankyrin repeat domain 1 (cardiac muscle)
A_21_P0000813	APELA	apelin receptor early endogenous ligand
A_22_P00002896	APELA	apelin receptor early endogenous ligand
A_22_P00002897	APELA	apelin receptor early endogenous ligand
A_33_P6462199	APELA	apelin receptor early endogenous ligand
A_23_P318860	APLNR	apelin receptor
A_24_P416961	ARVCF	armadillo repeat gene deleted in velocardiofacial syndrome
A_33_P3236993	ARVCF	armadillo repeat gene deleted in velocardiofacial syndrome
A_23_P216094	ASPH	aspartate beta-hydroxylase
A_24_P18105	ASPH	aspartate beta-hydroxylase
A_24_P295245	ASPH	aspartate beta-hydroxylase
A_24_P128145	ATF2	activating transcription factor 2
A_24_P246467	ATF2	activating transcription factor 2
A_23_P1072	ATP1A1	ATPase, Na+/K+ transporting, alpha 1 polypeptide
A_33_P3384272	ATP1A1	ATPase, Na+/K+ transporting, alpha 1 polypeptide
A_23_P148879	ATP1A2	ATPase, Na+/K+ transporting, alpha 2 polypeptide
A_33_P3359771	ATP1A2	ATPase, Na+/K+ transporting, alpha 2 polypeptide
A_33_P3283669	ATP1A3	ATPase, Na+/K+ transporting, alpha 3 polypeptide
A_23_P160177	ATP1A4	ATPase, Na+/K+ transporting, alpha 4 polypeptide
A_24_P307626	ATP1A4	ATPase, Na+/K+ transporting, alpha 4 polypeptide
A_23_P62932	ATP1B1	ATPase, Na+/K+ transporting, beta 1 polypeptide
A_24_P31275	ATP1B2	ATPase, Na+/K+ transporting, beta 2 polypeptide
A_23_P68007	ATP1B3	ATPase, Na+/K+ transporting, beta 3 polypeptide
A_23_P72462	ATP2A1	ATPase, Ca++ transporting, cardiac muscle, fast twitch 1
A_33_P3342720	ATP2A1	ATPase, Ca++ transporting, cardiac muscle, fast twitch 1
A_33_P3342830	ATP2A1	ATPase, Ca++ transporting, cardiac muscle, fast twitch 1
A_24_P141786	ATP2A2	ATPase, Ca++ transporting, cardiac muscle, slow twitch 2
A_24_P73290	ATP2A2	ATPase, Ca++ transporting, cardiac muscle, slow twitch 2
A_23_P207632	ATP2A3	ATPase, Ca++ transporting, ubiquitous
A_24_P202319	ATP2A3	ATPase, Ca++ transporting, ubiquitous
A_33_P3267185	ATP2B1	ATPase, Ca++ transporting, plasma membrane 1
A_33_P3267186	ATP2B1	ATPase, Ca++ transporting, plasma membrane 1
A_23_P18152	ATP2B2	ATPase, Ca++ transporting, plasma membrane 2
A_33_P3393851	ATP2B2	ATPase, Ca++ transporting, plasma membrane 2
A_33_P3400973	ATP2B2	ATPase, Ca++ transporting, plasma membrane 2
A_23_P337642	ATP2B3	ATPase, Ca++ transporting, plasma membrane 3
A_33_P3369146	ATP2B3	ATPase, Ca++ transporting, plasma membrane 3
A_23_P11841	ATP2B4	ATPase, Ca++ transporting, plasma membrane 4
A_24_P405205	ATP2B4	ATPase, Ca++ transporting, plasma membrane 4
A_33_P3327200	ATP2B4	ATPase, Ca++ transporting, plasma membrane 4
A_23_P213385	BASP1	brain abundant, membrane attached signal protein 1
A_23_P215449	BAZ1B	bromodomain adjacent to zinc finger domain, 1B
A_33_P3321577	BAZ1B	bromodomain adjacent to zinc finger domain, 1B
A_23_P99967	BBS4	Bardet-Biedl syndrome 4
A_23_P252642	BBS5	Bardet-Biedl syndrome 5
A_23_P5785	BBS5	Bardet-Biedl syndrome 5
A_23_P252913	BBS7	Bardet-Biedl syndrome 7
A_23_P159741	BCOR	BCL6 corepressor
A_23_P405707	BCOR	BCL6 corepressor
A_33_P3267760	BCOR	BCL6 corepressor
A_23_P131588	BMP10	bone morphogenetic protein 10
A_33_P3237150	BMP2	bone morphogenetic protein 2
A_23_P54144	BMP4	bone morphogenetic protein 4
A_19_P00805548	BMPR1A	bone morphogenetic protein receptor, type IA
A_23_P1431	BMPR1A	bone morphogenetic protein receptor, type IA
A_24_P527404	BMPR1A	bone morphogenetic protein receptor, type IA
A_33_P3219256	BMPR1A	bone morphogenetic protein receptor, type IA
A_32_P181638	BVES	blood vessel epicardial substance
A_32_P468341	C1orf127	chromosome 1 open reading frame 127
A_24_P371399	C3orf58	chromosome 3 open reading frame 58
A_23_P373031	CACNA1C	calcium channel, voltage-dependent, L type, alpha 1C subunit
A_33_P3249394	CACNA1C	calcium channel, voltage-dependent, L type, alpha 1C subunit
A_33_P3297580	CACNA1C	calcium channel, voltage-dependent, L type, alpha 1C subunit
A_33_P3825869	CACNA1C	calcium channel, voltage-dependent, L type, alpha 1C subunit
A_23_P365767	CACNA1D	calcium channel, voltage-dependent, L type, alpha 1D subunit
A_23_P148327	CACNA1F	calcium channel, voltage-dependent, L type, alpha 1F subunit
A_23_P107247	CACNA1G	calcium channel, voltage-dependent, T type, alpha 1G subunit
A_33_P3261418	CACNA1G	calcium channel, voltage-dependent, T type, alpha 1G subunit
A_33_P3218960	CACNA1H	calcium channel, voltage-dependent, T type, alpha 1H subunit
A_23_P85765	CACNA1S	calcium channel, voltage-dependent, L type, alpha 1S subunit
A_23_P82379	CACNA2D1	calcium channel, voltage-dependent, alpha 2/delta subunit 1
A_33_P3232478	CACNA2D1	calcium channel, voltage-dependent, alpha 2/delta subunit 1
A_23_P346900	CACNA2D2	calcium channel, voltage-dependent, alpha 2/delta subunit 2
A_24_P402825	CACNA2D3	calcium channel, voltage-dependent, alpha 2/delta subunit 3
A_33_P3373259	CACNA2D3	calcium channel, voltage-dependent, alpha 2/delta subunit 3
A_23_P353014	CACNA2D4	calcium channel, voltage-dependent, alpha 2/delta subunit 4

Systematic	Common	Description
A_33_P3285275	CACNB2	calcium channel, voltage-dependent, beta 2 subunit
A_33_P3285277	CACNB2	calcium channel, voltage-dependent, beta 2 subunit
A_33_P3363271	CACNB2	calcium channel, voltage-dependent, beta 2 subunit
A_23_P204016	CACNB3	calcium channel, voltage-dependent, beta 3 subunit
A_33_P3221989	CACNB4	calcium channel, voltage-dependent, beta 4 subunit
A_23_P89302	CACNG1	calcium channel, voltage-dependent, gamma subunit 1
A_22_P00003226	CACNG2	calcium channel, voltage-dependent, gamma subunit 2
A_23_P84000	CACNG2	calcium channel, voltage-dependent, gamma subunit 2
A_23_P66180	CACNG3	calcium channel, voltage-dependent, gamma subunit 3
A_24_P70303	CACNG4	calcium channel, voltage-dependent, gamma subunit 4
A_33_P3341299	CACNG4	calcium channel, voltage-dependent, gamma subunit 4
A_23_P414328	CACNG5	calcium channel, voltage-dependent, gamma subunit 5
A_23_P501933	CACNG6	calcium channel, voltage-dependent, gamma subunit 6
A_23_P4782	CACNG7	calcium channel, voltage-dependent, gamma subunit 7
A_24_P365349	CACNG7	calcium channel, voltage-dependent, gamma subunit 7
A_23_P420218	CACNG8	calcium channel, voltage-dependent, gamma subunit 8
A_24_P313186	CALM1	calmodulin 1 (phosphorylase kinase, delta)
A_23_P144458	CAMK2D	calcium/calmodulin-dependent protein kinase II delta
A_32_P47988	CAMK2D	calcium/calmodulin-dependent protein kinase II delta
A_23_P365189	CAMTA2	calmodulin binding transcription activator 2
A_23_P12572	CASP7	caspase 7, apoptosis-related cysteine peptidase
A_23_P173	CASQ1	calsequestrin 1 (fast-twitch, skeletal muscle)
A_23_P126582	CASQ2	calsequestrin 2 (cardiac muscle)
A_23_P134454	CAV1	caveolin 1, caveolae protein, 22kDa
A_24_P12626	CAV1	caveolin 1, caveolae protein, 22kDa
A_24_P251599	CAV3	caveolin 3
A_24_P74571	CBY1	chibby homolog 1 (Drosophila)
A_23_P26928	CCDC103	coiled-coil domain containing 103
A_33_P3257594	CCDC103	coiled-coil domain containing 103
A_33_P3315949	CCDC103	coiled-coil domain containing 103
A_32_P404549	CCDC39	coiled-coil domain containing 39
A_24_P160680	CCDC40	coiled-coil domain containing 40
A_33_P3302518	CCDC40	coiled-coil domain containing 40
A_23_P115645	CELF2	CUGBP, Elav-like family member 2
A_23_P202071	CELF2	CUGBP, Elav-like family member 2
A_24_P289383	CHD7	chromodomain helicase DNA binding protein 7
A_33_P3293164	CHGA	chromogranin A (parathyroid secretory protein 1)
A_23_P145606	CHRM2	cholinergic receptor, muscarinic 2
A_33_P3351298	CHRM2	cholinergic receptor, muscarinic 2
A_23_P214969	CITED2	Cbp/p300-interacting transactivator, with Glu/Asp-rich carboxy-terminal domain, 2
A_33_P3213374	CITED2	Cbp/p300-interacting transactivator, with Glu/Asp-rich carboxy-terminal domain, 2
A_23_P138760	CLCF1	cardiotrophin-like cytokine factor 1
A_33_P3285540	CLDN5	claudin 5
A_33_P3775848	CLIC2	chloride intracellular channel 2
A_23_P124946	CMYA5	cardiomyopathy associated 5
A_24_P935491	COL3A1	collagen, type III, alpha 1
A_23_P81131	CORIN	corin, serine peptidase
A_23_P259442	CPE	carboxypeptidase E
A_23_P155376	CRELD1	cysteine-rich with EGF-like domains 1
A_33_P3422479	CRELD1	cysteine-rich with EGF-like domains 1
A_23_P44674	CRIP1	cysteine-rich protein 1 (intestinal)
A_33_P3251703	CRIP1	cysteine-rich protein 1 (intestinal)
A_33_P3354965	CRIP1	cysteine-rich protein 1 (intestinal)
A_33_P3502315	CRLS1	cardiolipin synthase 1
A_23_P24469	CSRP3	cysteine and glycine-rich protein 3 (cardiac LIM protein)
A_33_P3822503	CTF1	cardiotrophin 1
A_24_P153831	CTNNA3	catenin (cadherin-associated protein), alpha 3
A_33_P3302191	CTNNA3	catenin (cadherin-associated protein), alpha 3
A_23_P29495	CTNNB1	catenin (cadherin-associated protein), beta 1, 88kDa
A_33_P3421695	CTNNB1	catenin (cadherin-associated protein), beta 1, 88kDa
A_23_P57268	CXADR	coxsackie virus and adenovirus receptor
A_24_P374943	CXADR	coxsackie virus and adenovirus receptor
A_23_P103486	CYP2J2	cytochrome P450, family 2, subfamily J, polypeptide 2
A_23_P435636	DAND5	DAN domain family member 5, BMP antagonist
A_23_P28466	DAW1	dynein assembly factor with WDR repeat domains 1
A_23_P98645	DCHS1	dachsous cadherin-related 1
A_33_P3275801	DES	desmin
A_23_P24129	DKK1	dickkopf WNT signaling pathway inhibitor 1
A_23_P112016	DLC1	DLC1 Rho GTPase activating protein
A_24_P940115	DLC1	DLC1 Rho GTPase activating protein
A_23_P167920	DLL1	delta-like 1 (Drosophila)
A_23_P419641	DLL4	delta-like 4 (Drosophila)
A_23_P321860	DMD	dystrophin
A_24_P185854	DMD	dystrophin
A_33_P3284763	DMD	dystrophin
A_33_P3297813	DMD	dystrophin
A_23_P50535	DMPK	dystrophia myotonica-protein kinase
A_23_P54612	DNAAF1	dynein, axonemal, assembly factor 1
A_33_P3368388	DNAAF1	dynein, axonemal, assembly factor 1
A_23_P255876	DNAI1	dynein, axonemal, intermediate chain 1
A_33_P3304242	DNAI1	dynein, axonemal, intermediate chain 1
A_33_P3733417	DRD2	dopamine receptor D2
A_22_P00005391	DSC2	desmocollin 2
A_23_P4494	DSC2	desmocollin 2
A_23_P141730	DSG2	desmoglein 2
A_32_P157945	DSP	desmoplakin
A_33_P3402565	DSP	desmoplakin
A_23_P139704	DUSP6	dual specificity phosphatase 6
A_23_P55342	DVL2	disheveled segment polarity protein 2
A_24_P154080	ECE1	endothelin converting enzyme 1
A_24_P396375	ECE1	endothelin converting enzyme 1
A_23_P377299	ECE2	endothelin converting enzyme 2
A_23_P92261	ECE2	endothelin converting enzyme 2
A_23_P214821	EDN1	endothelin 1
A_33_P3258392	EDN1	endothelin 1
A_23_P312150	EDN2	endothelin 2
A_23_P17438	EDN3	endothelin 3
A_33_P3397288	EDN3	endothelin 3
A_23_P113005	EFNA1	ephrin-A1
A_24_P355944	EFNB2	ephrin-B2
A_23_P83328	ENG	endoglin
A_23_P168443	EPHB4	EPH receptor B4
A_33_P3216532	EPHB4	EPH receptor B4
A_23_P349416	ERBB3	erb-b2 receptor tyrosine kinase 3
A_33_P3211569	ERBB3	erb-b2 receptor tyrosine kinase 3
A_33_P3280471	ERBB3	erb-b2 receptor tyrosine kinase 3
A_32_P183765	ERBB4	erb-b2 receptor tyrosine kinase 4
A_33_P3211558	ERBB4	erb-b2 receptor tyrosine kinase 4
A_24_P62783	FABP3	fatty acid binding protein 3, muscle and heart
A_33_P3348239	FBN1	fibrillin 1

Systematic	Common	Description
A_33_P3379886	FGF2	fibroblast growth factor 2 (basic)
A_33_P3419696	FGF2	fibroblast growth factor 2 (basic)
A_33_P3380797	FGF3	fibroblast growth factor 3
A_24_P355720	FGF4	fibroblast growth factor 4
A_23_P46829	FGF8	fibroblast growth factor 8 (androgen-induced)
A_23_P202334	FGFR2	fibroblast growth factor receptor 2
A_23_P303145	FGFR2	fibroblast growth factor receptor 2
A_24_P206624	FGFR2	fibroblast growth factor receptor 2
A_23_P397238	FKBP1A	FK506 binding protein 1A, 12kDa
A_24_P160001	FKBP1A	FK506 binding protein 1A, 12kDa
A_32_P50522	FKBP1A	FK506 binding protein 1A, 12kDa
A_33_P3227345	FKBP1A	FK506 binding protein 1A, 12kDa
A_33_P3403615	FKBP1A	FK506 binding protein 1A, 12kDa
A_23_P142631	FKBP1B	FK506 binding protein 1B, 12.6 kDa
A_24_P236235	FLRT2	fibronectin leucine rich transmembrane protein 2
A_33_P3418833	FLRT3	fibronectin leucine rich transmembrane protein 3
A_23_P53176	FOLR1	folate receptor 1 (adult)
A_32_P205110	FOXC1	forkhead box C1
A_33_P3302295	FOXC2	forkhead box C2
A_23_P118254	FOXF1	forkhead box F1
A_33_P3355503	FOXL1	forkhead box L1
A_32_P140030	FOXN4	forkhead box N4
A_23_P155257	FOXP1	forkhead box P1
A_33_P3214303	FOXP1	forkhead box P1
A_33_P3214310	FOXP1	forkhead box P1
A_33_P3228435	FXYD1	FXYD domain containing ion transport regulator 1
A_22_P00025271	FXYD2	FXYD domain containing ion transport regulator 2
A_23_P161769	FXYD2	FXYD domain containing ion transport regulator 2
A_33_P3228445	FXYD2	FXYD domain containing ion transport regulator 2
A_33_P3228450	FXYD3	FXYD domain containing ion transport regulator 3
A_33_P3228455	FXYD3	FXYD domain containing ion transport regulator 3
A_33_P3228460	FXYD3	FXYD domain containing ion transport regulator 3
A_33_P3228466	FXYD3	FXYD domain containing ion transport regulator 3
A_23_P98121	FXYD4	FXYD domain containing ion transport regulator 4
A_23_P150394	FXYD6	FXYD domain containing ion transport regulator 6
A_23_P119611	FXYD7	FXYD domain containing ion transport regulator 7
A_23_P153026	GAA	glucosidase, alpha; acid
A_23_P8416	GALNT11	polypeptide N-acetylgalactosaminyltransferase 11
A_24_P374244	GATA1	GATA binding protein 1 (globin transcription factor 1)
A_33_P3550894	GATA2	GATA binding protein 2
A_33_P3360341	GATA3	GATA binding protein 3
A_23_P384761	GATA4	GATA binding protein 4
A_33_P3293456	GATA4	GATA binding protein 4
A_33_P3360363	GATA4	GATA binding protein 4
A_33_P3406962	GATA4	GATA binding protein 4
A_23_P371835	GATA5	GATA binding protein 5
A_23_P304450	GATA6	GATA binding protein 6
A_24_P55295	GJA1	gap junction protein, alpha 1, 43kDa
A_23_P371729	GJA5	gap junction protein, alpha 5, 40kDa
A_33_P3373358	GJC1	gap junction protein, gamma 1, 45kDa
A_23_P209246	GLI2	GLI family zinc finger 2
A_33_P3358457	GLI2	GLI family zinc finger 2
A_33_P3358462	GLI2	GLI family zinc finger 2
A_33_P3358469	GLI2	GLI family zinc finger 2
A_24_P381029	GLRX3	glutaredoxin 3
A_23_P318284	GPD1L	glycerol-3-phosphate dehydrogenase 1-like
A_23_P8640	GPER1	G protein-coupled estrogen receptor 1
A_33_P3345812	GPER1	G protein-coupled estrogen receptor 1
A_33_P3345816	GPER1	G protein-coupled estrogen receptor 1
A_33_P3356752	GPER1	G protein-coupled estrogen receptor 1
A_33_P3239849	GPX1	glutathione peroxidase 1
A_33_P3354322	GPX1	glutathione peroxidase 1
A_23_P432947	GREM1	gremlin 1, DAN family BMP antagonist
A_33_P3319870	GREM1	gremlin 1, DAN family BMP antagonist
A_24_P40626	GREM2	gremlin 2, DAN family BMP antagonist
A_24_P185394	GSK3A	glycogen synthase kinase 3 alpha
A_23_P397208	GSTM2	glutathione S-transferase mu 2 (muscle)
A_33_P3410351	GSTM2	glutathione S-transferase mu 2 (muscle)
A_24_P304051	GSTO1	glutathione S-transferase omega 1
A_23_P58770	HAND1	heart and neural crest derivatives expressed 1
A_23_P373521	HAND2	heart and neural crest derivatives expressed 2
A_23_P10206	HAS2	hyaluronan synthase 2
A_33_P3418681	HCN4	hyperpolarization activated cyclic nucleotide gated potassium channel 4
A_33_P3418686	HCN4	hyperpolarization activated cyclic nucleotide gated potassium channel 4
A_23_P210048	HDAC4	histone deacetylase 4
A_22_P00007581	HDAC9	histone deacetylase 9
A_23_P404162	HDAC9	histone deacetylase 9
A_24_P206317	HDAC9	histone deacetylase 9
A_32_P166693	HEG1	heart development protein with EGF-like domains 1
A_23_P6596	HES1	hes family bHLH transcription factor 1
A_32_P83845	HEY1	hes-related family bHLH transcription factor with YRPW motif 1
A_24_P363408	HEY2	hes-related family bHLH transcription factor with YRPW motif 2
A_23_P430658	HEYL	hes-related family bHLH transcription factor with YRPW motif-like
A_23_P142125	HRC	histidine rich calcium binding protein
A_23_P103703	HSPB7	heat shock 27kDa protein family, member 7 (cardiovascular)
A_33_P3368369	HSPB7	heat shock 27kDa protein family, member 7 (cardiovascular)
A_23_P16953	HTR2B	5-hydroxytryptamine (serotonin) receptor 2B, G protein-coupled
A_23_P212447	IFT122	intraflagellar transport 122
A_33_P3338698	IHH	indian hedgehog
A_23_P35684	INPP5F	inositol polyphosphate-5-phosphatase F
A_33_P3237096	INPP5F	inositol polyphosphate-5-phosphatase F
A_33_P3238920	INPP5F	inositol polyphosphate-5-phosphatase F
A_33_P3335725	INSR	insulin receptor
A_23_P110837	IRX4	iroquois homeobox 4
A_23_P81529	ISL1	ISL LIM homeobox 1
A_23_P92042	ITPR1	inositol 1,4,5-trisphosphate receptor, type 1
A_22_P00008337	ITPR2	inositol 1,4,5-trisphosphate receptor, type 2
A_33_P3232294	ITPR2	inositol 1,4,5-trisphosphate receptor, type 2
A_33_P3298128	ITPR2	inositol 1,4,5-trisphosphate receptor, type 2
A_23_P19517	ITPR3	inositol 1,4,5-trisphosphate receptor, type 3
A_23_P210763	JAG1	jagged 1
A_23_P501822	JUP	junction plakoglobin
A_23_P417173	KCNA5	potassium channel, voltage gated shaker related subfamily A, member 5
A_23_P315772	KCND1	potassium channel, voltage gated Shal related subfamily D, member 1
A_23_P259251	KCND2	potassium channel, voltage gated Shal related subfamily D, member 2
A_23_P692	KCND3	potassium channel, voltage gated Shal related subfamily D, member 3
A_32_P58407	KCND3	potassium channel, voltage gated Shal related subfamily D, member 3
A_23_P154855	KCNE1	potassium channel, voltage gated subfamily E regulatory beta subunit 1
A_23_P120694	KCNE2	potassium channel, voltage gated subfamily E regulatory beta subunit 2
A_23_P256641	KCNE5	potassium channel, voltage gated subfamily E regulatory beta subunit 5

Systematic	Common	Description
A_23_P168403	KCNH2	potassium channel, voltage gated eag related subfamily H, member 2
A_23_P377882	KCNH2	potassium channel, voltage gated eag related subfamily H, member 2
A_23_P30554	KCNIP1	Kv channel interacting protein 1
A_24_P12998	KCNIP2	Kv channel interacting protein 2
A_23_P356004	KCNIP3	Kv channel interacting protein 3, calsenilin
A_33_P3274194	KCNIP4	Kv channel interacting protein 4
A_23_P1973	KCNJ11	potassium channel, inwardly rectifying subfamily J, member 11
A_24_P339429	KCNJ12	potassium channel, inwardly rectifying subfamily J, member 12
A_23_P130764	KCNJ14	potassium channel, inwardly rectifying subfamily J, member 14
A_23_P329261	KCNJ2	potassium channel, inwardly rectifying subfamily J, member 2
A_24_P6125	KCNJ4	potassium channel, inwardly rectifying subfamily J, member 4
A_23_P126075	KCNK1	potassium channel, two pore domain subfamily K, member 1
A_33_P3358277	KCNK1	potassium channel, two pore domain subfamily K, member 1
A_22_P00008538	KCNK15	potassium channel, two pore domain subfamily K, member 15
A_23_P109026	KCNK15	potassium channel, two pore domain subfamily K, member 15
A_23_P91104	KCNK3	potassium channel, two pore domain subfamily K, member 3
A_23_P50591	KCNK6	potassium channel, two pore domain subfamily K, member 6
A_23_P429977	KCNQ1	potassium channel, voltage gated KQT-like subfamily Q, member 1
A_33_P3247331	KCNQ1	potassium channel, voltage gated KQT-like subfamily Q, member 1
A_24_P942630	KDM6B	lysine (K)-specific demethylase 6B
A_33_P3382217	KIF3A	kinesin family member 3A
A_33_P3465247	KIF3A	kinesin family member 3A
A_23_P120227	LBH	limb bud and heart development
A_33_P3364263	LBH	limb bud and heart development
A_33_P3364268	LBH	limb bud and heart development
A_24_P66337	LCLAT1	lysocardiolipin acyltransferase 1
A_32_P92505	LCLAT1	lysocardiolipin acyltransferase 1
A_33_P3349693	LCLAT1	lysocardiolipin acyltransferase 1
A_23_P160336	LEFTY1	left-right determination factor 1
A_22_P00004121	lnc-CLCNKB-4	lnc-CLCNKB-4:1
A_22_P00007040	lnc-GJA5-2	lnc-GJA5-2:1
A_22_P00009494	lnc-MAGI1-2	lnc-MAGI1-2:1
A_22_P00011847	lnc-PHOX2A-1	lnc-PHOX2A-1:1
A_22_P00015791	lnc-TARBP2-1	lnc-TARBP2-1:1
A_22_P00018011	lnc-ZNF18-1	lnc-ZNF18-1:1
A_22_P00018366	lnc-ZWINT-5	lnc-ZWINT-5:1
A_33_P3335800	LOC100128219	uncharacterized LOC100128219
A_33_P3306948	LRP6	low density lipoprotein receptor-related protein 6
A_33_P3388865	LRRC10	leucine rich repeat containing 10
A_23_P110606	MAML1	mastermind-like 1 (Drosophila)
A_33_P3220149	MAML1	mastermind-like 1 (Drosophila)
A_33_P3341676	MEF2A	myocyte enhancer factor 2A
A_24_P393470	MEF2BNB	MEF2B neighbor
A_24_P914940	MEF2BNB	MEF2B neighbor
A_33_P3354771	MEF2BNB	MEF2B neighbor
A_23_P320739	MEF2C	myocyte enhancer factor 2C
A_23_P51679	MEF2D	myocyte enhancer factor 2D
A_33_P3341656	MEF2D	myocyte enhancer factor 2D
A_33_P3715177	MEGF8	multiple EGF-like-domains 8
A_33_P3214466	MESP1	mesoderm posterior basic helix-loop-helix transcription factor 1
A_23_P13442	MICAL2	microtubule associated monooxygenase, calponin and LIM domain containing 2
A_33_P3315190	MICAL2	microtubule associated monooxygenase, calponin and LIM domain containing 2
A_33_P3279550	MIXL1	Mix paired-like homeobox
A_22_P00009972	MKKS	McKusick-Kaufman syndrome
A_23_P79962	MKKS	McKusick-Kaufman syndrome
A_33_P3388135	MKKS	McKusick-Kaufman syndrome
A_23_P24211	MMP21	matrix metallopeptidase 21
A_23_P111492	MOSPD3	motile sperm domain containing 3
A_33_P3321946	MOSPD3	motile sperm domain containing 3
A_23_P117494	MTHFD1	methylenetetrahydrofolate dehydrogenase (NADP+ dependent) 1, methenyltetrahydrofolate cyclohydrolase, formyltetrahydrofol
A_23_P127385	MYBPC3	myosin binding protein C, cardiac
A_23_P206920	MYH11	myosin, heavy chain 11, smooth muscle
A_24_P70183	MYH11	myosin, heavy chain 11, smooth muscle
A_21_P0013396	MYH6	myosin, heavy chain 6, cardiac muscle, alpha
A_23_P37167	MYH6	myosin, heavy chain 6, cardiac muscle, alpha
A_24_P282383	MYH7	myosin, heavy chain 7, cardiac muscle, beta
A_33_P3266964	MYH7	myosin, heavy chain 7, cardiac muscle, beta
A_23_P162547	MYL2	myosin, light chain 2, regulatory, cardiac, slow
A_24_P131646	MYL3	myosin, light chain 3, alkali; ventricular, skeletal, slow
A_24_P151032	MYL4	myosin, light chain 4, alkali; atrial, embryonic
A_23_P80008	MYLK2	myosin light chain kinase 2
A_22_P00010389	MYLK3	myosin light chain kinase 3
A_33_P3300217	MYLK3	myosin light chain kinase 3
A_24_P53241	MYOCD	myocardin
A_33_P3259042	MYOCD	myocardin
A_23_P162228	NACA	nascent polypeptide-associated complex alpha subunit
A_22_P00010556	NCOA6	nuclear receptor coactivator 6
A_23_P259135	NCOA6	nuclear receptor coactivator 6
A_23_P140748	NDRG4	NDRG family member 4
A_22_P00001222	NDUFV2	NADH dehydrogenase (ubiquinone) flavoprotein 2, 24kDa
A_23_P130418	NDUFV2	NADH dehydrogenase (ubiquinone) flavoprotein 2, 24kDa
A_24_P398147	NEBL	nebulette
A_33_P3244007	NEBL	nebulette
A_33_P3244013	NEBL	nebulette
A_24_P108311	NEDD4L	neural precursor cell expressed, developmentally down-regulated 4-like, E3 ubiquitin protein ligase
A_24_P782308	NEDD4L	neural precursor cell expressed, developmentally down-regulated 4-like, E3 ubiquitin protein ligase
A_33_P3391005	NEDD4L	neural precursor cell expressed, developmentally down-regulated 4-like, E3 ubiquitin protein ligase
A_23_P200001	NEXN	nexilin (F actin binding protein)
A_33_P3341429	NEXN	nexilin (F actin binding protein)
A_21_P0012236	NF1	neurofibromin 1
A_24_P1919	NF1	neurofibromin 1
A_24_P917026	NF1	neurofibromin 1
A_33_P3240348	NF1	neurofibromin 1
A_23_P213883	NIPBL	Nipped-B homolog (Drosophila)
A_33_P3299599	NKX2-5	NK2 homeobox 5
A_33_P3240632	NKX2-6	NK2 homeobox 6
A_33_P3391796	NOG	noggin
A_23_P204791	NOS1	nitric oxide synthase 1 (neuronal)
A_33_P3365167	NOS1	nitric oxide synthase 1 (neuronal)
A_23_P74309	NOS1AP	nitric oxide synthase 1 (neuronal) adaptor protein
A_23_P60387	NOTCH1	notch 1
A_33_P3370424	NOTCH1	notch 1
A_23_P200792	NOTCH2	notch 2
A_33_P3258824	NOTCH2	notch 2
A_33_P3264771	NOTO	notochord homeobox
A_33_P3228072	NPHP3	nephronophthisis 3 (adolescent)
A_33_P3228102	NPHP3	nephronophthisis 3 (adolescent)
A_23_P74059	NPPA	natriuretic peptide A
A_33_P3323699	NPPA	natriuretic peptide A
A_23_P62752	NPPB	natriuretic peptide B

Systematic	Common	Description
A_23_P301942	NPPC	natriuretic peptide C
A_22_P00015046	NPR1	natriuretic peptide receptor 1
A_23_P147711	NPR1	natriuretic peptide receptor 1
A_23_P123622	NPR2	natriuretic peptide receptor 2
A_23_P69699	NPY1R	neuropeptide Y receptor Y1
A_23_P58169	NPY2R	neuropeptide Y receptor Y2
A_33_P3396244	NPY2R	neuropeptide Y receptor Y2
A_23_P251836	NPY5R	neuropeptide Y receptor Y5
A_23_P315815	NRG1	neuregulin 1
A_33_P3284345	NRG1	neuregulin 1
A_24_P135322	NRP1	neuropilin 1
A_24_P928052	NRP1	neuropilin 1
A_23_P393727	NRP2	neuropilin 2
A_24_P50801	NRP2	neuropilin 2
A_33_P3297415	NRP2	neuropilin 2
A_23_P205900	NTRK3	neurotrophic tyrosine kinase, receptor, type 3
A_32_P141969	NTRK3	neurotrophic tyrosine kinase, receptor, type 3
A_33_P3260134	NTRK3	neurotrophic tyrosine kinase, receptor, type 3
A_33_P3322814	NTRK3	neurotrophic tyrosine kinase, receptor, type 3
A_23_P300484	OBSL1	obscurin-like 1
A_33_P3241428	OBSL1	obscurin-like 1
A_24_P406601	OLFM1	olfactomedin 1
A_33_P3248354	OLFM1	olfactomedin 1
A_33_P3306749	OLFM1	olfactomedin 1
A_23_P323272	OSR1	odd-skipped related transciption factor 1
A_33_P3362088	P2RX4	purinergic receptor P2X, ligand gated ion channel, 4
A_24_P124370	PARVA	parvin, alpha
A_23_P257003	PCSK5	proprotein convertase subtilisin/kexin type 5
A_33_P3358731	PCSK5	proprotein convertase subtilisin/kexin type 5
A_33_P3358735	PCSK5	proprotein convertase subtilisin/kexin type 5
A_23_P74278	PDE4B	phosphodiesterase 4B, cAMP-specific
A_33_P3240552	PDE4D	phosphodiesterase 4D, cAMP-specific
A_33_P3389649	PDE4D	phosphodiesterase 4D, cAMP-specific
A_33_P3389653	PDE4D	phosphodiesterase 4D, cAMP-specific
A_33_P3389658	PDE4D	phosphodiesterase 4D, cAMP-specific
A_24_P339944	PDGFB	platelet-derived growth factor beta polypeptide
A_23_P300033	PDGFRA	platelet-derived growth factor receptor, alpha polypeptide
A_22_P00011688	PDGFRB	platelet-derived growth factor receptor, beta polypeptide
A_23_P421401	PDGFRB	platelet-derived growth factor receptor, beta polypeptide
A_32_P14610	PDLIM5	PDZ and LIM domain 5
A_33_P3278664	PDLIM5	PDZ and LIM domain 5
A_33_P3336925	PDLIM5	PDZ and LIM domain 5
A_33_P3416479	PHKG1	phosphorylase kinase, gamma 1 (muscle)
A_21_P0012238	PIK3CA	phosphatidylinositol-4,5-bisphosphate 3-kinase, catalytic subunit alpha
A_23_P92057	PIK3CA	phosphatidylinositol-4,5-bisphosphate 3-kinase, catalytic subunit alpha
A_33_P3304170	PIK3CG	phosphatidylinositol-4,5-bisphosphate 3-kinase, catalytic subunit gamma
A_23_P167367	PITX2	paired-like homeodomain 2
A_21_P0011419	PKD1	polycystic kidney disease 1 (autosomal dominant)
A_23_P77502	PKD1	polycystic kidney disease 1 (autosomal dominant)
A_33_P3812815	PKD1	polycystic kidney disease 1 (autosomal dominant)
A_24_P106112	PKD2	polycystic kidney disease 2 (autosomal dominant)
A_33_P3405728	PKP2	plakophilin 2
A_23_P35617	PLCE1	phospholipase C, epsilon 1
A_33_P3346669	PLCE1	phospholipase C, epsilon 1
A_24_P414803	PLN	phospholamban
A_23_P167017	POPDC2	popeye domain containing 2
A_24_P257336	POPDC2	popeye domain containing 2
A_23_P59138	POU5F1	POU class 5 homeobox 1
A_24_P144601	POU5F1	POU class 5 homeobox 1
A_23_P329375	POU6F1	POU class 6 homeobox 1
A_23_P408285	PRICKLE1	prickle homolog 1 (Drosophila)
A_24_P399630	PRKACA	protein kinase, cAMP-dependent, catalytic, alpha
A_24_P88266	PROX1	prospero homeobox 1
A_33_P3311974	PROX2	prospero homeobox 2
A_33_P3362178	PROX2	prospero homeobox 2
A_23_P98085	PTEN	phosphatase and tensin homolog
A_24_P913115	PTEN	phosphatase and tensin homolog
A_23_P22096	PTK2	protein tyrosine kinase 2
A_23_P105436	PTPN11	protein tyrosine phosphatase, non-receptor type 11
A_23_P99027	PTPN11	protein tyrosine phosphatase, non-receptor type 11
A_24_P116710	RAMP2	receptor (G protein-coupled) activity modifying protein 2
A_23_P111737	RAMP3	receptor (G protein-coupled) activity modifying protein 3
A_23_P254498	RANGRF	RAN guanine nucleotide release factor
A_23_P55136	RANGRF	RAN guanine nucleotide release factor
A_24_P453497	RBM20	RNA binding motif protein 20
A_33_P3307157	RBM20	RNA binding motif protein 20
A_33_P3307163	RBM20	RNA binding motif protein 20
A_23_P75283	RBP4	retinol binding protein 4, plasma
A_21_P0013567	RBPJ	recombination signal binding protein for immunoglobulin kappa J region
A_23_P29994	RBPJ	recombination signal binding protein for immunoglobulin kappa J region
A_23_P167081	REST	RE1-silencing transcription factor
A_23_P114947	RGS2	regulator of G-protein signaling 2
A_33_P3304533	RNF207	ring finger protein 207
A_33_P3304538	RNF207	ring finger protein 207
A_33_P3316978	RNF207	ring finger protein 207
A_23_P80503	ROBO1	roundabout, axon guidance receptor, homolog 1 (Drosophila)
A_23_P78867	RYR1	ryanodine receptor 1 (skeletal)
A_23_P137797	RYR2	ryanodine receptor 2 (cardiac)
A_33_P3251727	RYR2	ryanodine receptor 2 (cardiac)
A_33_P3800734	RYR3	ryanodine receptor 3
A_23_P383227	S100A1	S100 calcium binding protein A1
A_33_P3409086	S100A1	S100 calcium binding protein A1
A_23_P404481	S1PR1	sphingosine-1-phosphate receptor 1
A_23_P328074	SALL1	spalt-like transcription factor 1
A_23_P158555	SCN10A	sodium channel, voltage gated, type X alpha subunit
A_23_P79015	SCN1B	sodium channel, voltage gated, type I beta subunit
A_32_P187571	SCN2B	sodium channel, voltage gated, type II beta subunit
A_33_P3394699	SCN2B	sodium channel, voltage gated, type II beta subunit
A_33_P3402404	SCN3B	sodium channel, voltage gated, type III beta subunit
A_23_P303833	SCN4B	sodium channel, voltage gated, type IV beta subunit
A_23_P21063	SCN5A	sodium channel, voltage gated, type V alpha subunit
A_24_P128233	SCN5A	sodium channel, voltage gated, type V alpha subunit
A_33_P3350547	SCN5A	sodium channel, voltage gated, type V alpha subunit
A_33_P3391455	SCN5A	sodium channel, voltage gated, type V alpha subunit
A_33_P3230990	SCUBE1	signal peptide, CUB domain, EGF-like 1
A_33_P3417487	SCUBE1	signal peptide, CUB domain, EGF-like 1
A_33_P3480395	SCUBE1	signal peptide, CUB domain, EGF-like 1
A_33_P3299510	SCX	scleraxis basic helix-loop-helix transcription factor
A_23_P341532	SETDB2	SET domain, bifurcated 2
A_33_P3327245	SETDB2	SET domain, bifurcated 2

Systematic	Common	Description
A_32_P4595	SGCD	sarcoglycan, delta (35kDa dystrophin-associated glycoprotein)
A_33_P3267375	SGCD	sarcoglycan, delta (35kDa dystrophin-associated glycoprotein)
A_33_P3267380	SGCD	sarcoglycan, delta (35kDa dystrophin-associated glycoprotein)
A_23_P423237	SGCG	sarcoglycan, gamma (35kDa dystrophin-associated glycoprotein)
A_23_P373584	SGCZ	sarcoglycan, zeta
A_32_P24585	SH3PXD2B	SH3 and PX domains 2B
A_23_P111657	SHH	sonic hedgehog
A_33_P3221859	SHOX2	short stature homeobox 2
A_23_P132121	SIK1	salt-inducible kinase 1
A_24_P136866	SLC8A1	solute carrier family 8 (sodium/calcium exchanger), member 1
A_32_P147078	SLC8A1	solute carrier family 8 (sodium/calcium exchanger), member 1
A_33_P3262156	SLC8A1	solute carrier family 8 (sodium/calcium exchanger), member 1
A_33_P3319542	SLC8A2	solute carrier family 8 (sodium/calcium exchanger), member 2
A_24_P167654	SLC8A3	solute carrier family 8 (sodium/calcium exchanger), member 3
A_24_P350683	SLC9A1	solute carrier family 9, subfamily A (NHE1, cation proton antiporter 1), member 1
A_24_P71373	SLC9A1	solute carrier family 9, subfamily A (NHE1, cation proton antiporter 1), member 1
A_33_P3267059	SLC9A1	solute carrier family 9, subfamily A (NHE1, cation proton antiporter 1), member 1
A_23_P55518	SMAD7	SMAD family member 7
A_23_P70818	SMO	smoothened, frizzled class receptor
A_33_P3379816	SMYD1	SET and MYND domain containing 1
A_23_P131846	SNAI1	snail family zinc finger 1
A_23_P169039	SNAI2	snail family zinc finger 2
A_24_P322709	SNTA1	syntrophin, alpha 1
A_23_P154840	SOD1	superoxide dismutase 1, soluble
A_23_P134176	SOD2	superoxide dismutase 2, mitochondrial
A_23_P121795	SORBS2	sorbin and SH3 domain containing 2
A_24_P140475	SORBS2	sorbin and SH3 domain containing 2
A_33_P3406861	SORBS2	sorbin and SH3 domain containing 2
A_24_P302584	SOX11	SRY (sex determining region Y)-box 11
A_23_P82775	SOX17	SRY (sex determining region Y)-box 17
A_33_P3226761	SOX18	SRY (sex determining region Y)-box 18
A_24_P911676	SOX4	SRY (sex determining region Y)-box 4
A_23_P26847	SOX9	SRY (sex determining region Y)-box 9
A_24_P238420	SPTBN4	spectrin, beta, non-erythrocytic 4
A_24_P293114	SPTBN4	spectrin, beta, non-erythrocytic 4
A_33_P3401459	SPTBN4	spectrin, beta, non-erythrocytic 4
A_23_P2414	SPX	spexin hormone
A_23_P322086	SPX	spexin hormone
A_23_P308603	SRC	SRC proto-oncogene, non-receptor tyrosine kinase
A_24_P337657	SRF	serum response factor (c-fos serum response element-binding transcription factor)
A_23_P59718	SRI	sorcin
A_23_P314755	STC1	stanniocalcin 1
A_24_P214231	STIL	SCL/TAL1 interrupting locus
A_33_P3400477	STIL	SCL/TAL1 interrupting locus
A_23_P53162	STIM1	stromal interaction molecule 1
A_33_P3307495	STRA6	stimulated by retinoic acid 6
A_33_P3307500	STRA6	stimulated by retinoic acid 6
A_33_P3709150	T	T, brachyury homolog (mouse)
A_22_P00015733	TAB2	TGF-beta activated kinase 1/MAP3K7 binding protein 2
A_23_P19702	TAB2	TGF-beta activated kinase 1/MAP3K7 binding protein 2
A_33_P3404331	TAB2	TGF-beta activated kinase 1/MAP3K7 binding protein 2
A_23_P62371	TAZ	tafazzin
A_33_P3409077	TBC1D32	TBC1 domain family, member 32
A_23_P211345	TBX1	T-box 1
A_23_P40527	TBX1	T-box 1
A_23_P164451	TBX2	T-box 2
A_24_P211849	TBX20	T-box 20
A_23_P25176	TBX5	T-box 5
A_24_P30557	TBX5	T-box 5
A_23_P107051	TCAP	titin-cap
A_23_P49220	TCF25	transcription factor 25 (basic helix-loop-helix)
A_24_P306561	TCF25	transcription factor 25 (basic helix-loop-helix)
A_23_P366376	TDGF1	teratocarcinoma-derived growth factor 1
A_23_P374695	TEK	TEK tyrosine kinase, endothelial
A_24_P79054	TGFB1	transforming growth factor, beta 1
A_24_P402438	TGFB2	transforming growth factor, beta 2
A_33_P3331451	TGFBR1	transforming growth factor, beta receptor 1
A_23_P211957	TGFBR2	transforming growth factor, beta receptor II (70/80kDa)
A_33_P3313825	TGFBR2	transforming growth factor, beta receptor II (70/80kDa)
A_23_P200780	TGFBR3	transforming growth factor, beta receptor III
A_33_P3252414	TH	tyrosine hydroxylase
A_33_P3360997	TH	tyrosine hydroxylase
A_33_P3494748	TMEM65	transmembrane protein 65
A_23_P166823	TNNC1	troponin C type 1 (slow)
A_23_P51565	TNNI1	troponin I type 1 (skeletal, slow)
A_33_P3362831	TNNI1	troponin I type 1 (skeletal, slow)
A_23_P24784	TNNI2	troponin I type 2 (skeletal, fast)
A_23_P67453	TNNI3	troponin I type 3 (cardiac)
A_24_P415680	TNNI3K	TNNI3 interacting kinase
A_23_P34700	TNNT2	troponin T type 2 (cardiac)
A_24_P413470	TP73	tumor protein p73
A_33_P3296831	TP73	tumor protein p73
A_23_P206018	TPM1	tropomyosin 1 (alpha)
A_24_P44462	TPM1	tropomyosin 1 (alpha)
A_33_P3335966	TPM1	tropomyosin 1 (alpha)
A_33_P3248799	TRDN	triadin
A_24_P28977	TRPC1	transient receptor potential cation channel, subfamily C, member 1
A_23_P153529	TRPM4	transient receptor potential cation channel, subfamily M, member 4
A_23_P134755	TRPS1	trichorhinophalangeal syndrome I
A_33_P3361746	TRPS1	trichorhinophalangeal syndrome I
A_23_P66110	TSC2	tuberous sclerosis 2
A_33_P3281686	TSC2	tuberous sclerosis 2
A_23_P68219	TTN	titin
A_23_P85269	TTN	titin
A_33_P3329428	TTN	titin
A_33_P3329433	TTN	titin
A_21_P0005474	TWIST1	twist family bHLH transcription factor 1
A_21_P0005597	TWIST1	twist family bHLH transcription factor 1
A_23_P71073	TWIST1	twist family bHLH transcription factor 1
A_33_P3414591	TWIST1	twist family bHLH transcription factor 1
A_33_P3282005	VANGL2	VANGL planar cell polarity protein 2
A_23_P70398	VEGFA	vascular endothelial growth factor A
A_24_P12401	VEGFA	vascular endothelial growth factor A
A_23_P411157	WNT1	wingless-type MMTV integration site family, member 1
A_33_P3382588	WNT1	wingless-type MMTV integration site family, member 1
A_23_P102117	WNT10A	wingless-type MMTV integration site family, member 10A
A_23_P162322	WNT10B	wingless-type MMTV integration site family, member 10B
A_24_P253003	WNT11	wingless-type MMTV integration site family, member 11
A_23_P134601	WNT16	wingless-type MMTV integration site family, member 16
A_23_P59807	WNT2	wingless-type MMTV integration site family member 2

Systematic	Common	Description
A_23_P130158	WNT3	wingless-type MMTV integration site family, member 3
A_23_P385690	WNT3A	wingless-type MMTV integration site family, member 3A
A_33_P3417502	WNT3A	wingless-type MMTV integration site family, member 3A
A_33_P3321070	WNT4	wingless-type MMTV integration site family, member 4
A_33_P3397525	WNT4	wingless-type MMTV integration site family, member 4
A_33_P3341499	WNT5A	wingless-type MMTV integration site family, member 5A
A_23_P53588	WNT5B	wingless-type MMTV integration site family, member 5B
A_23_P119916	WNT6	wingless-type MMTV integration site family, member 6
A_23_P258410	WNT7A	wingless-type MMTV integration site family, member 7A
A_24_P911607	WNT7B	wingless-type MMTV integration site family, member 7B
A_33_P3248903	WNT7B	wingless-type MMTV integration site family, member 7B
A_33_P3256920	WNT7B	wingless-type MMTV integration site family, member 7B
A_23_P58854	WNT8A	wingless-type MMTV integration site family, member 8A
A_23_P52430	WNT8B	wingless-type MMTV integration site family, member 8B
A_33_P3384058	WNT9A	wingless-type MMTV integration site family, member 9A
A_23_P89587	WNT9B	wingless-type MMTV integration site family, member 9B
A_23_P116280	WT1	Wilms tumor 1
A_23_P108743	XIRP2	xin actin-binding repeat containing 2
A_23_P393163	XIRP2	xin actin-binding repeat containing 2
A_24_P33444	YWHAE	tyrosine 3-monooxygenase/tryptophan 5-monooxygenase activation protein, epsilon
A_33_P3407636	YWHAE	tyrosine 3-monooxygenase/tryptophan 5-monooxygenase activation protein, epsilon
A_33_P3214720	ZC3H12A	zinc finger CCCH-type containing 12A
A_33_P3311979	ZFPM1	zinc finger protein, FOG family member 1
A_21_P0014737	ZFPM2	zinc finger protein, FOG family member 2
A_23_P168909	ZFPM2	zinc finger protein, FOG family member 2
A_23_P327910	ZIC3	Zic family member 3
A_33_P3250861	ZIC3	Zic family member 3
